# Calreticulin as Cancer Treatment Adjuvant: Combination with Photodynamic Therapy and Photodynamic Therapy-Generated Vaccines

**DOI:** 10.3389/fonc.2015.00015

**Published:** 2015-02-03

**Authors:** Mladen Korbelik, Judit Banáth, Kyi Min Saw, Wei Zhang, Evaldas Čiplys

**Affiliations:** ^1^British Columbia Cancer Agency, Vancouver, BC, Canada; ^2^Vilnius University Institute of Biotechnology, Vilnius, Lithuania

**Keywords:** calreticulin, photodynamic therapy, cancer vaccine, DAMPs, antitumor-immune response

## Abstract

Calreticulin is recognized as one of the pivotal damage-associated molecular pattern molecules alerting the host of the presence of distressed cells. In this role, calreticulin becomes exposed on the surface of tumor cells treated by several types of cancer therapy including photodynamic therapy (PDT). The goal of the present study was to examine the potential of externally added calreticulin for augmenting antitumor effect mediated by PDT. Recombinant calreticulin was found to bind to mouse SCCVII tumor cells treated by PDT. Compared to the outcome with PDT alone, cure rates of SCCVII tumors grown in immunocompetent C3H/HeN mice were elevated when calreticulin (0.4 mg/mouse) was injected peritumorally immediately after PDT. Such therapeutic gain with PDT plus calreticulin combination was not obtained with SCCVII tumors growing in immunodeficient NOD-scid mice. In PDT-vaccine protocol, where PDT-treated SCCVII cells are used for vaccination of SCCVII tumor-bearing mice, adding recombinant calreticulin to cells before their injection produced improved therapeutic effect. The expression of calreticulin gene was reduced in PDT-treated cells, while no changes were observed with the expression of this gene in tumor, liver, and spleen tissues in PDT-vaccine-treated mice. These findings reveal that externally added recombinant calreticulin can boost antitumor response elicited by PDT or PDT-generated vaccines, and can thus serve as an effective adjuvant for cancer treatment with PDT and probably other cancer cell stress-inducing modalities.

## Introduction

Calreticulin is primarily ER-residing chaperone protein with a broad array of cellular functions, including maintenance of adequate calcium levels, protein folding and trafficking, gene transcription regulation, cell adherence and migration, apoptosis and dead cell clearance, and immune responses (1, 2). Cancer therapy-mediated lethal insults that involve stress induction in the ER induce the translocation of calreticulin to the outer leaflet of the plasma membrane by an active process occurring before the appearance of morphological signs of apoptosis (3, 4). Such surface-exposed calreticulin serves as a powerful mobilizing signal to the immune system, which has inspired recognition of calreticulin as one of the most important damage-associated molecular patterns (DAMPs) (5). As danger signals, DAMPs alert the host that immune response is required to restore homeostasis (3, 6). Surface calreticulin expression on dying cancer cells is critical for their demise by immunological cell death (ICD) resulting in immune rejection of tumors with the same cells (7).

Photodynamic therapy (PDT) belongs to cancer modalities capable of inducing an abundance of DAMPs including surface-exposed calreticulin (5, 8). This therapy is established clinically for treatment of various malignant and non-oncological lesions (9). It works by localized generation of reactive oxygen species mediated by the transfer of energy absorbed by light-activated drugs (photosensitizers) to molecular oxygen (10, 11). Photooxidative lesions induced in PDT-treated cells provoke a strong oxidative stress that with many photosensitizers includes the ER. Such a type of stress was suggested to lead to the escape of calreticulin and other ER molecules to the cell surface (4). Ceramide and sphingosine-1-phosphate are ER-derived molecules that were likewise found exposed on the surface of PDT-treated cells and were also shown to act as DAMPs (12, 13). With PDT mediated by photosensitizer chlorin e6 (ce6), we detected cell surface-exposed calreticulin as early as 15 min post-treatment (Korbelik, unpublished).

Abundant engagement after PDT of various DAMPs, both cancer cell surface expressed and released from treated cells was suggested as one of the key elements responsible for the capacity of this modality to elicit a strong immune response against treated tumor (14). This PDT-induced antitumor-immune response can be enhanced by a variety of immunostimulating treatments for achieving superior tumor cures (15). The present report describes how such approach can include recombinant calreticulin protein. The research questions to be addressed were: first, can the recombinant calreticulin be used as an effective adjuvant to PDT and/or PDT-generated vaccine therapy? Second, what investigative directives should be followed when designing research for the comprehensive elucidation of the underlying mechanism of action?

## Materials and Methods

### Tumor model

The squamous cell carcinoma model SCCVII syngeneic to C3H/HeN mice, a recognized model of head and neck cancer of spontaneous origin with absence of strong immunogenicity (16), was implanted subcutaneously into lower dorsal site of 7–9-week-old C3H/HeN or NOD-scid mice. The tumors were treated with PDT vaccine when they reached 5 mm and with PDT *in situ* at 7–8 mm size in largest diameter. The procedures used with mice were in compliance with the protocols approved by the Animal Care Committee of the University of British Columbia. Cultures of SCCVII cells were maintained in alpha minimal essential medium supplemented with 10% fetal bovine serum (Life Technologies, Burlington, ON, Canada).

### Preparation of recombinant human calreticulin

Native recombinant human calreticulin was generated using yeast as expression host based on the methodology developed in previous work (17, 18). Whole coding sequence of human calreticulin gene (GenBank accession no. M84739) was PCR-cloned from human adult liver cDNA library (Clontech Laboratories, Saint-Germain-en-Laye, France) using primers that generate *Xba*I restriction sites. The PCR product was digested and cloned into the *Xma*JI site of pPIC3.5K shuttle vector (Invitrogen, Life Technologies) under control of *Pichia pastoris* AOX1 promoter. The pPIC3.5K-CRT plasmid was transformed into *P. pastoris* GS115 (*his4*) (Invitrogen) by electroporation after linearization with *Bgl*II restriction endonuclease. Several multi-copy integrants grown on various geneticin concentrations were screened for secretion of human calreticulin. Recombinant *P. pastoris* strain (GS115:hCRT) with the optimal secretion of human calreticulin was chosen for further studies. It was initially grown in the Ygly medium (2% peptone from soybean, 1% yeast extract, 1% glycerin, 2 × 10^−5^% biotin). After 24 h incubation, the cells were transferred into Ymet medium (2% peptone from soybean, 1% yeast extract, 1% methanol, 2 × 10^−5^% biotin) and incubated for 120 h, with 1% methanol supplanted every 8 h. Following this induction period, cells were separated from the medium by centrifugation at 10,000 *g* for 20 min. Yeast growth medium was subjected to prefiltration and microfiltration through filters with pore size of 1.6, 0.45, and 0.2 μM (cat. nos. FT-3-1101-047, 15406-47 and 15407-47-MIN, respectively, SartoriusStedim Biotech, Goettingen, Germany). After microfiltration, proteins were concentrated and transferred into the binding buffer (20 mM l-histidine, pH 5.5, 100 mM NaCl) through tangential ultrafiltration using cassettes with 100 kDa cut-off membranes (cat. no.VF20P3, SartoriusStedim Biotech). Protein sample was loaded onto the Q Sepharose FastFlow column (cat. no. 17-0510-10, GE Healthcare, Freiburg, Germany). Unbound proteins were washed from the column with 10 column volumes of binding buffer, while bound proteins were eluted with 15 column volumes NaCl gradient (100–500 mM). In the final step, the elution fractions were analyzed by SDS-PAGE, and the fractions showing ≥ 90% pure human calreticulin were pooled and dialyzed against storage buffer (20 mM Tris-HCl, pH 7.5, 100 mM NaCl, 3 mM CaCl_2_). The SDS-PAGE gel with the final calreticulin preparation used in the experiments with cells and *in vivo* is shown in Figure 2.

### PDT and PDT-vaccine treatment

Two different treatments involving photosensitizing drug exposure followed by light application were performed using photosensitizers Temoporfin for PDT and chlorin e6 (ce6) for PDT vaccines in standard doses established in previous extensive investigations. The protocol used for PDT-generated vaccine treatment was described in detail earlier (19, 20). Briefly, for each treatment group (6 mice), 1.2 × 10^8^ SCCVII cells were exposed to ce6 (purchased from Frontier Scientific Inc., Logan, UT, USA) for 30 min at 0.5 μg/ml followed by 1 J/cm^2^ of 665 ± 10 nm light. The light, delivered through a liquid light guide (model 77638, Oriel Instruments, Stratford, CT, USA), was produced by a 150 W QTH lamp-based high-throughput source with an integrated ellipsoid reflector and interchangeable interference filters (model FB-QTH-3, Sciencetech Inc., London, ON, Canada). This PDT treatment was lethal for around 70% of cells, most of them dying by apoptosis. After PDT treatment, the cells were left overnight (16 h) at 37°C in EX-CELL chemically defined protein- and serum-free medium (Sigma Chemical Co., St. Louis, MO, USA). The cells were then collected, concentrated by centrifugation for treatment with X-rays (60 Gy) followed in some treatment groups by the exposure to recombinant rabbit calreticulin (Abcam Inc., Cambridge, MA, USA) at the concentration of 15 μg per vaccine dose for 30 min on ice. The vaccine cells were then used immediately for peritumoral injection (2 × 10^7^ per mouse). As a part of the routine PDT-vaccine protocol, mice received low-dose cyclophosphamide (50 mg/kg i.p.) 24 h before vaccination (for controlling immunoregulatory cell activity). The therapeutic effect of PDT vaccines was assessed by monitoring changes in tumor size.

For PDT treatment of SCCVII tumors, the host mice were administered Temoporfin (active pharmaceutical ingredient of Foscan, provided by Biolitec Research GmbH, Jena, Germany) at the dose of 0.1 mg/kg i.p., and 24 h later, the tumors were exposed superficially to 655 ± 10 nm light (using the source described above) with a dose rate of 80–90 mW/cm^2^ while the mice were immobilized in specially designed holders. The mice were subsequently monitored for tumor growth and those showing no sign of palpable tumor at 90 days post-treatment qualified as cured. For testing the effect of PDT plus calreticulin combination, each mouse was injected peritumorally 400 μg of recombinant human calreticulin contained in 0.1 ml PBS immediately after photodynamic light treatment.

### Calreticulin association with PDT-treated cells

The protocol was identical as used with the PDT-generated vaccine (exposure of SCCVII cells to ce6-based PDT followed by 16 h post-incubation and then 30 min exposure to calreticulin on ice), except that the recombinant human calreticulin was used and its concentration was 20 μg per million cells. In one treatment group, mitoxantrone (M6545, Sigma) 0.5 μg/ml was present during the 16-h post-incubation period. Presence of calreticulin on the surface of SCCVII cells was assessed by staining the cells with chicken polyclonal antibody to calreticulin (reactive with both human and mouse protein) followed by Alexa Fluor 488-conjugated goat anti-chicken IgY both purchased from Abcam. For isotype control, ChromPure chicken IgY (Jackson ImmunoResearch Laboratories, West Grove, PA, USA) was used as the primary antibody. Suspensions of stained cells were then analyzed by flow cytometry performed on Coulter Epics Elite ESP (Coulter Electronics, Hialeah, FL, USA) with 20,000 cells measured in each test. Fluorescence values per cell obtained with the isotype control were deducted from values recorded with the calreticulin antibody.

### Calreticulin gene expression analysis

Total RNA was extracted from SCCVII cells or mice tissues using Trizol and cleaned up with Qiagen MinElute (Qiagen Canada Inc., Montreal QC, Canada). It served for performing gene expression analysis as described in detail in our previous reports (20, 21). Briefly, the extracted total RNA was used for creating complementary strand DNA transcript that was then amplified by quantitative RT-PCR in the presence of mouse calreticulin gene (NCBI Reference Sequence NM_007591.3)-specific primers CTGAATACAAGGGCGAGTGGA (forward) and GCATCGGGGGAGTATTCAGG (reverse) that were designed and tested in our laboratory. The expression of housekeeping gene glycerladehide-3-phosphate dehydrogenase (GAPDH) was also measured and used for normalizing the expression of calreticulin gene.

### Statistical analysis

Each experiment was repeated at least once. Statistical evaluation of tumor response to PDT or PDT-vaccine treatment was done using log-rank test, while the analysis of other data was based on Mann–Whitney test with the threshold for statistical significance set at 5%.

## Results

**Figure 1:**
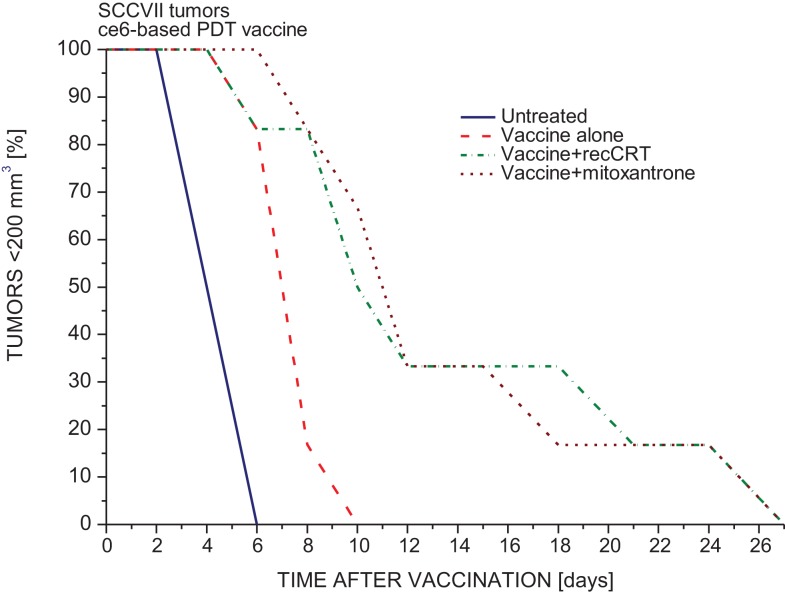
**The impact of calreticulin exposure or mitoxantrone treatment on the potency of PDT-generated vaccine**. PDT vaccine was prepared by treating SCCVII cells with ce6-PDT (30 min exposure to 0.5 μg/ml ce6 followed by 1 J/cm^2^ of 665 ± 10 nm light) and leaving them in culture for 16 h post-treatment incubation before they were injected (20 million/mouse) peritumorally into C3H/HeN mice bearing SCCVII tumors. With one treatment group, mitoxantrone (0.5 μg/ml) was present during the post-treatment incubation. Before injection, vaccine cells for one group of mice were exposed to recombinant rabbit calreticulin (15 μg per 20 million cells) for 30 min on ice. For other details of PDT vaccine protocol, Section “Materials and Methods.” Each treatment group consisted of 6 mice. The response, based on two experiments, of SCCVII tumors to vaccine treatment is depicted as changes in the percentage of mice with tumors smaller than 200 mm^3^ in time after therapy. Statistically significant difference (*p* < 0.05): untreated vs. vaccine alone groups, vaccine alone vs. vaccine with calreticulin groups, and vaccine vs. vaccine with mitoxantrone.

The PDT-generated vaccine protocol with SCCVII tumor model developed in our laboratory, centers on preparing single-cell cancer vaccines by using *in vitro* PDT-treated SCCVII cells post-incubated in culture for 16 h and then injected peritumorally into mice bearing SCCVII tumors (19). To determine whether the efficacy of PDT vaccine can be affected by modifying calreticulin involvement, before injecting them to mice the vaccine cells were altered either by 30 min exposure to calreticulin protein or by treatment with mitoxantrone (known to induce cell surface expression of calreticulin (22). The results are presented in Figure 1. Compared to untreated SCCVII tumors, PDT vaccine alone treatment produced a significant retardation in the growth rate of these relatively fast growing tumors. This therapeutic gain was further improved by both adjuvant calreticulin (15 μg per dose) and the exposure to mitoxantrone during the post-PDT incubation of vaccine cells (Figure 1). Thus, the time for all the tumors to reach the critical size of 200 mm^3^ was extended from 10 to 27 days. The same calreticulin dose per mouse was used by Obeid et al. (22). Using the vaccine SCCVII cells treated with calreticulin or mitoxantrone alone without PDT had no significant impact on tumor growth rates compared to those with untreated tumors (not shown).

**Figure 2:**
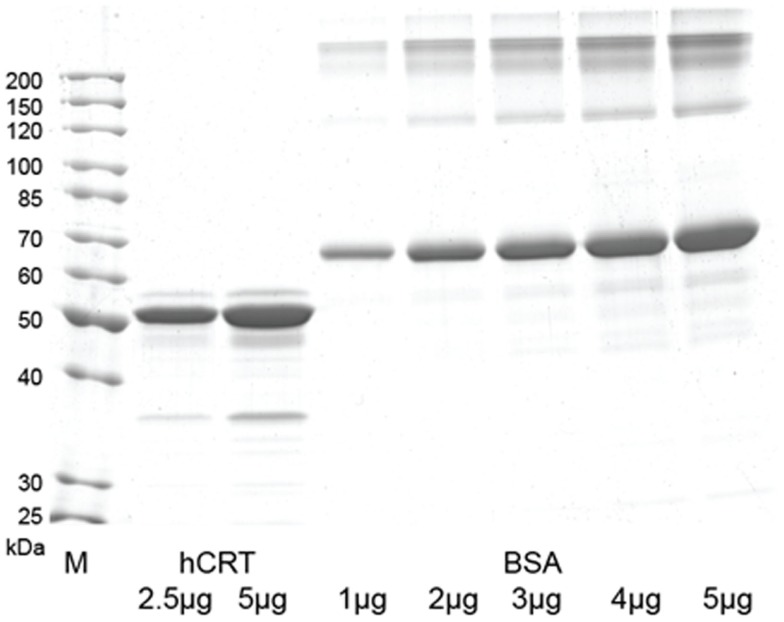
**SDS-PAGE gel with human calreticulin protein purified from yeast culture medium**. The gel was loaded with 2.5 and 5 μg of human calreticulin and 1–5 μg of BSA (cat. no. B14, Thermo Fisher Scientific, Waltham, MA, USA) as amount control. M – Unstained protein ladder (cat. no. 26614, Thermo Fisher Scientific).

**Figure 3:**
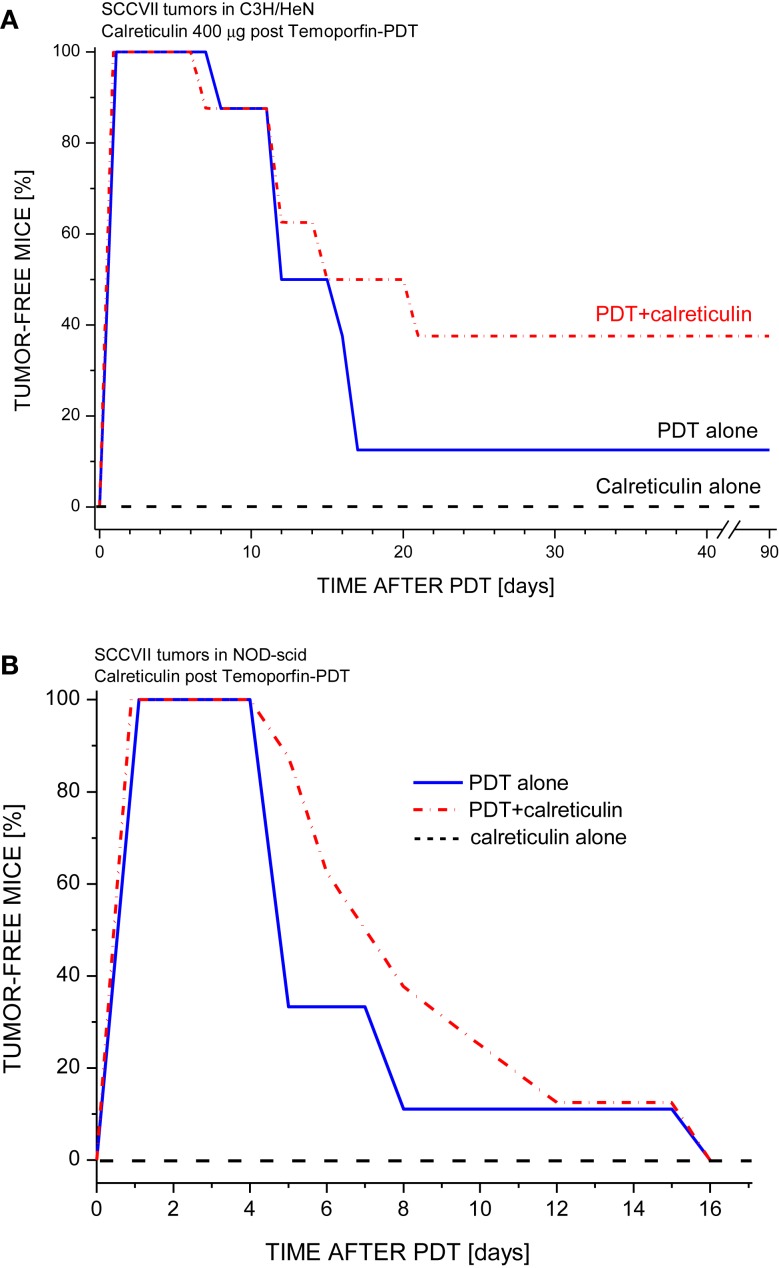
**The effect of tumor-localized calreticulin injection on PDT response of SCCVII tumors growing in immunocompetent or immunodeficient mice**. The tumors growing either in **(A)** syngeneic immunocompetent mice (C3H/HeN) or **(B)** in immunodeficient mice (NOD-scid) were treated by Temoporfin-PDT (0.1 mg/kg i.p. Temoporfin followed 24 h later by 80 J/cm^2^ light dose). Recombinant human calreticulin (400 μg/mouse) was injected peritumorally immediately after PDT light. Mice were then monitored for up to 90 days for signs of tumor regrowth. Each treatment group consisted of 8 mice. With C3H/HeN mice, the response of PDT alone and PDT plus calreticulin groups were statistically different (*p* < 0.05).

The finding that calreticulin can act as an effective adjuvant to PDT vaccine encouraged our further investigation for which we secured required quantities of recombinant calreticulin by developing its production from yeast carrying human calreticulin gene. The SDS-PAGE gel with the final calreticulin preparation used in the experiments with cells and *in vivo* is shown in Figure 2. This allowed us to examine the effects of calreticulin on the response of tumors treated *in situ* by PDT. For this study, SCCVII tumors were implanted subcutaneously either into immunocompetent syngeneic C3H/HeN mice or into immunodeficient NOD-scid mice. When tumors reached 7–8 mm in largest diameter, the mice were administered photosensitizer Temoporfin, and 24 h later, the tumors were exposed to PDT illumination. Since recombinant human calreticulin was administered directly into mice immediately after light treatment, its dose was escalated to 400 μg per injection. All PDT-treated tumors became impalpable and necrotic within 24 h but recurrence was detected with many mice within 2 weeks post-therapy. The results with immunocompetent mice (Figure 3A) showed that the adjuvant calreticulin treatment rendered a significant improvement in tumor response to PDT from marginally curative to the solid levels of about 40% cure rates. There was no evidence with PDT-treated tumors of any effect following peritumoral injection of the same volume of saline (not shown). No significant effect on tumor was observed following treatment with calreticulin alone. In immunodeficient mice, the adjuvant calreticulin was not effective in improving tumor response to PDT (Figure 3B) and again no effect was observed with calreticulin alone.

**Figure 4:**
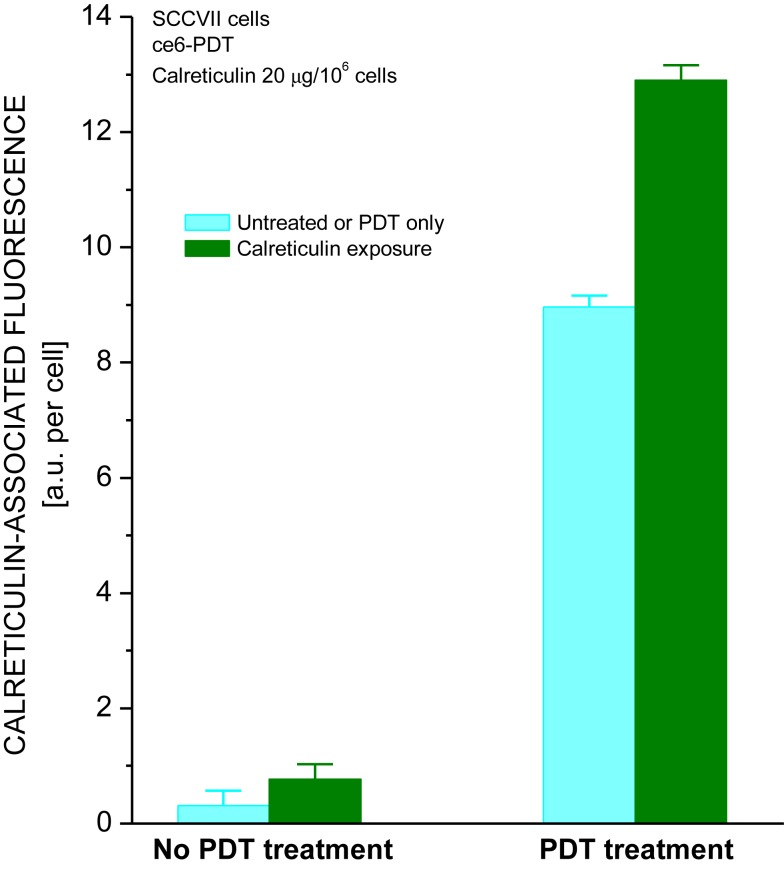
**Calreticulin binds to PDT-treated cells**. The treatment of SCCVII cells with ce6-PDT and 16-h post-incubation was performed using the protocol for PDT vaccine preparation described in Figure 1. The cells were then concentrated by centrifugation and kept on ice in the presence of recombinant human calreticulin (20 μg per million cells). Cell suspensions were then stained with chicken antibody raised against calreticulin or with isotype control immunoglobulin, followed by Alexa Fluor 488-conjugated secondary antibody, and analyzed by flow cytometry. The data show the levels of cell surface-localized calreticulin on untreated and PDT-treated cells that were either unexposed or exposed to recombinant calreticulin. *N* = 4, bars are SD, *statistically significant difference (*p* < 0.05) compared to calreticulin unexposed PDT-treated cells.

One of the proteins sharing with calreticulin the capacity to act as DAMP is heat shock protein 70 (Hsp70) (5, 23). We have shown that externally supplied Hsp70 protein binds to tumor cells that have sustained oxidative stress mediated by PDT (24). To test whether a similar property is exhibited by calreticulin, mouse tumor SCCVII cells were treated *in vitro* by PDT using photosensitizer ce6. After 16-h post-PDT culture at 37°C, the cells were transferred on ice and further incubated 30 min with recombinant human calreticulin added to the medium. The calreticulin dose range was 1–20 μg per10^6^ cells, i.e., similar to the levels described for *in vitro* experiments by Obeid et al. (22). The cells were then stained with anti-calreticulin antibody for detecting the presence of this protein on the surface of cells using flow cytometry. The results show that the calreticulin protein present in the medium binds to PDT-treated cells but not to untreated cells (Figure 4). As shown before (8, 12), PDT treatment induced the presentation of endogenous calreticulin on the surface of cells. However, the calreticulin levels on these cells were significantly greater when in contact with the externally added calreticulin. Binding of this protein to PDT-treated cells could not be detected when less than 1 μg per million cells was added to the medium (not shown).

**Figure 5:**
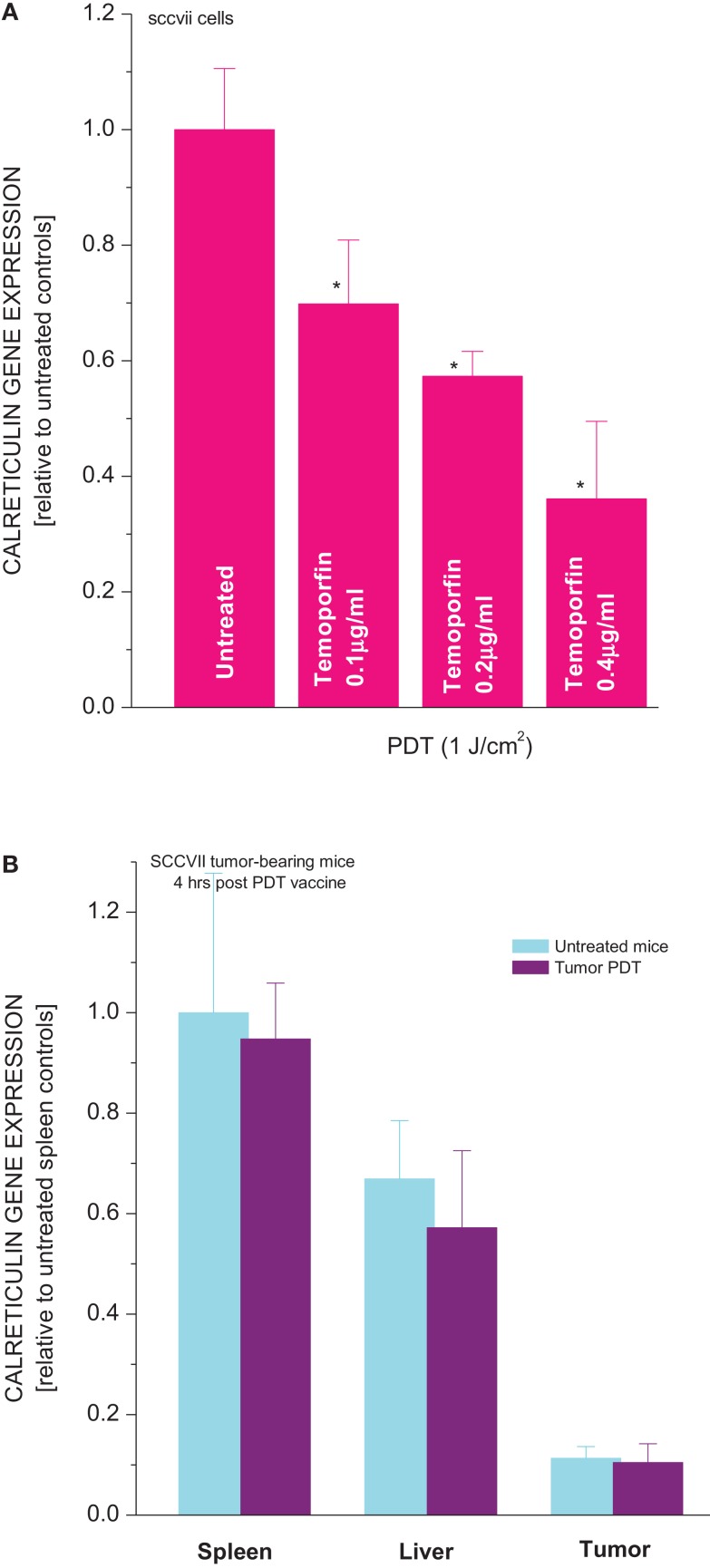
**Expression of calreticulin gene in PDT-treated cells and tumor, liver, and spleen tissues of mice bearing PDT-treated SCCVII tumors**. In experiments *in vitro* **(A)** SCCVII cells were exposed to Temoporfin (0.1, 0.2, or 0.4 μg/ml for 24 h) and then treated with 655 ± 10 nm light (1 J/cm^2^). The cells were collected 3 h after PDT and their total RNA isolated. In experiments *in vivo* **(B)** C3H/HeN mice with SCCVII tumors received tumor-localized PDT vaccine treatment as described for Figure 1. The mice were sacrificed 4 h later and their tumor, liver, and spleen tissues collected for total RNA isolation. The obtained total RNA samples were used for quantitative RT-PCR-based analysis of the expression of calreticulin gene. The results are presented as GAPDH-normalized mouse calreticulin gene expression relative to the same feature in untreated cells or in the same tissue of untreated tumor-bearing mice. *N* = 4, bars are SD, *statistically significant difference (*p* < 0.05) compared to calreticulin gene expression in PDT-untreated cells.

Another characteristic of Hsp70 is that its gene becomes upregulated not only in tumor cells directly treated by PDT but also in the livers and spleens of mice with PDT-treated or PDT vaccine-treated tumors (20, 24). In contrast, the expression of gene encoding calreticulin decreased in SCCVII cells treated by PDT (Figure 5A). The effect was PDT dose dependent, with close to 50% reduction found in cells 3 h after they received the medium tested PDT dose that attains around 90% cell kill. *In vivo*, no significant differences were found in the calreticulin gene expression in tumor, liver, and spleen tissues between untreated and PDT vaccine-treated mice (Figure 5B).

## Discussion

In one of the earliest reports on the therapy-induced translocation of calreticulin to the surface of tumor cells and its relevance, Obeid et al. (22) demonstrated that anthracyclines or their analog mitoxantrone are effective in achieving an such effect and that this is responsible for ICD produced by these drugs. In the same work, the authors showed that tumor cells treated by a chemotherapy agent mitoxantrone not only translocated intracellular calreticulin to their surface but were also able to bind externally added calreticulin protein. In accordance to these findings, our previous investigation revealed the induction of surface calreticulin expression on SCCVII cells treated with this neoplastic agent as well as with PDT mediated by photosensitizer Photofrin (12), while the present study shows that calreticulin protein present in cell medium has no detectable affinity for binding to untreated cells but associates readily with PDT-treated tumor cells.

Our results demonstrate that this association with externally provided calreticulin renders PDT-vaccine cells therapeutically more potent (Figure 1). The similar benefit can be obtained by treating PDT-vaccine cells with mitoxantrone, which will further raise surface calreticulin levels originally prompted to emerge by PDT treatment. In the protocols for PDT of tumors *in situ*, the therapeutic gain seen with adjuvant calreticulin (Figure 3) can be expected to result from the association of this protein with cancer cells upon its administration by tumor-localized injection. Importantly, such therapeutic gain was not produced with the host mice deficient in mature lymphocytes (NOD-scid).

The capacity for binding to the surface of stressed tumor cells such as those treated by PDT calreticulin shares with another molecular chaperon and DAMP, hsp70 (24). Indeed, both of them could be binding to their common receptor CD91 on macrophages (25) but the identity of their binding partner on the surface of stressed tumor cells is not clear. Nonetheless, there are important differences between calreticulin and Hsp70. Unlike with calreticulin, tumor-localized recombinant Hsp70 protein injection after PDT has no impact on therapy outcome (Korbelik, unpublished results). On the other hand, as shown in Figure 5, calreticulin does not share with Hsp70 the capacity for gene expression and production to be upregulated in PDT-treated cells and be mobilized systemically as acute phase reactant, as evidenced by the upregulation of Hsp70 gene in the liver and spleen of mice bearing PDT-treated or a PDT vaccine-treated tumor (20, 24). Since the primary function of Hsp70 is expediting the repair or elimination of damaged intracellular proteins within stressed cells, it can be assumed that the cells will upgrade its gene while trying to survive. In contrast, the function of surface-exposed calreticulin is to facilitate the disposal of the cells doomed not to survive; in these cells, sufficient calreticulin can be supplied from the existing intracellular locations. This is in conformity with the finding that the surface exposure of calreticulin is entirely regulated in the cytoplasm by post-transcriptional and/or post-translational processes (22).

Calreticulin accumulated on the surface of tumor cells may modulate their death. Unlike Hsp70, which has inhibitory role in cell death pathways (26), the role of calreticulin is controversial due to reports suggesting its protection against apoptotic stimuli while other evidence infers its participation in the cell death pathway (4). The facts that tumor cells decorated with calreticulin are more efficiently phagocytosed by dendritic cells and have increased immunogenicity (22) are more consistent with the role of calreticulin as promoter of their death and disposal. Since surface-exposed calreticulin is one of the three DAMPs (together with ATP and HMGB1) attributed a key role in the immunogenic potential of ICD inducers (7), it seems rational to expect that its adjuvant properties described in the present study are also based on its role of promoting ICD. Calreticulin treatment adjuvant to PDT results in markedly increased levels of this protein exposed on the cell surface by adding from the external source to that of the internal origin already present. Evidently, the added calreticulin must become bound to certain (as yet unknown) molecules that appear on the surface of PDT-treated cells. This elevation of calreticulin levels on cancer cell surface appears to enhance its potential of promoting ICD, presumably by boosting their removal by antigen-presenting cells through phagocytosis pathways optimized for recognition of tumor antigens.

In summary, the present study demonstrates that externally added calreticulin protein, which has the ability to bind to tumor cells sustaining injury/stress from PDT treatment, can serve as an effective adjuvant augmenting tumor control with this therapy. Lack of such positive effect with tumors growing in immunodeficient mice indicates that the observed therapeutic gain with adjuvant calreticulin entails the engagement of adaptive immune system. The underlying mechanistic aspects remain to be uncovered in more detail with studies such as tumor immune infiltrate analysis. Based on the presented results, the hypothesis to be tested is that the adjuvant treatment with calreticulin provides a supplement DAMP material and promotes phagocytosis of PDT-treated tumor cells enhancing the development of antitumor-immune response (14, 22).

## Conflict of Interest Statement

The authors declare that the research was conducted in the absence of any commercial or financial relationships that could be construed as a potential conflict of interest.
